# Limitations of Artificial Intelligence-Based Tools in Psychotherapy

**DOI:** 10.1192/j.eurpsy.2025.222

**Published:** 2025-08-26

**Authors:** B. Ozel

**Affiliations:** Department of Psychiatry, Mus State Hospital, Mus, Türkiye

## Abstract

**Abstract:**

Artificial intelligence (AI) tools have gained attention for their potential to transform mental health care. However, in the context of psychotherapy, these tools reveal fundamental limitations that warrant critical analysis.

Psychotherapy is deeply rooted in relational dynamics, cultural sensitivity, and the ability to engage with the complex emotional landscapes of individuals. Current AI-based tools often struggle to address these dimensions adequately. While language models can generate coherent and seemingly empathetic responses, their inability to genuinely understand and respond to nuanced human emotions and interpersonal subtleties presents a significant barrier (Floridi & Chiriatti, 2020).

Ethical concerns also pose considerable challenges. The use of AI in psychotherapy raises questions about data privacy, algorithmic transparency, and the potential perpetuation of biases within AI systems. Such issues not only risk undermining the therapeutic process but may also erode trust in these technologies.

The therapeutic alliance—a critical factor in psychotherapy—represents another area of concern. Effective therapy relies heavily on building trust, mutual understanding, and a genuine connection, all of which are difficult to replicate with AI-based tools. Current research suggests that while AI can support certain therapeutic tasks, its capacity to maintain and enhance the therapeutic alliance remains unproven (Linardon et al., 2019).

This presentation will critically examine these limitations through case-based discussions and clinical reflections. By highlighting the inherent challenges of integrating AI into psychotherapy, it aims to encourage thoughtful and cautious exploration of its applications, emphasizing the importance of preserving the relational and ethical foundations of psychotherapeutic practice.

**Disclosure of Interest:**

None Declared

